# Diat.barcode, an open-access curated barcode library for diatoms

**DOI:** 10.1038/s41598-019-51500-6

**Published:** 2019-10-22

**Authors:** Frédéric Rimet, Evgenuy Gusev, Maria Kahlert, Martyn G. Kelly, Maxim Kulikovskiy, Yevhen Maltsev, David G. Mann, Martin Pfannkuchen, Rosa Trobajo, Valentin Vasselon, Jonas Zimmermann, Agnès Bouchez

**Affiliations:** 1 INRA, UMR CARRTEL, 75bis av. de Corzent - CS 50511, FR-74203 Thonon les Bains cedex, France; 2 University of Savoie Mont-Blanc, UMR CARRTEL, FR-73370 Le Bourget du Lac, France; 3 Institute of Plant Physiology, Russian Academy of Sciences, 127276 Moscow, Russia; 4 Swedish University of Agricultural Sciences, Department of Aquatic Sciences and Assessment, PO Box 7050, SE- 750 07 Uppsala, Sweden; 5 Bowburn Consultancy, 11 Monteigne Drive, Bowburn, Durham DH6 5QB UK; 6 Royal Botanic Garden Edinburgh, Edinburgh, EH3 5LR Scotland UK; 7 Marine and Continental Waters, Institute for Food and Agricultural Research and Technology (IRTA), Crta de Poble Nou Km 5.5, Sant Carles de la Ràpita, Catalonia Spain; 8 Institut Ruđer Bošković, Giordano Paliaga 5, 52210 Rovinj, Croatia; 9 Botanischer Garten und Botanisches Museum Berlin–Dahlem, Freie Universität Berlin, Königin–Luise–Str. 6–8, 14195 Berlin, Germany

**Keywords:** Molecular ecology, Molecular ecology, Taxonomy, Taxonomy, Genetic markers

## Abstract

Diatoms (Bacillariophyta) are ubiquitous microalgae which produce a siliceous exoskeleton and which make a major contribution to the productivity of oceans and freshwaters. They display a huge diversity, which makes them excellent ecological indicators of aquatic ecosystems. Usually, diatoms are identified using characteristics of their exoskeleton morphology. DNA-barcoding is an alternative to this and the use of High-Throughput-Sequencing enables the rapid analysis of many environmental samples at a lower cost than analyses under microscope. However, to identify environmental sequences correctly, an expertly curated reference library is needed. Several curated libraries for protists exists; none, however are dedicated to diatoms. Diat.barcode is an open-access library dedicated to diatoms which has been maintained since 2012. Data come from two sources (1) the NCBI nucleotide database and (2) unpublished sequencing data of culture collections. Since 2017, several experts have collaborated to curate this library for *rbc*L, a chloroplast marker suitable for species-level identification of diatoms. For the latest version of the database (version 7), 605 of the 3482 taxonomical names originally assigned by the authors of the *rbc*L sequences were modified after curation. The database is accessible at https://www6.inra.fr/carrtel-collection_eng/Barcoding-database.

## Introduction

The Bacillariophyta (diatoms) is a particularly species diverse phylum of microalgae with an estimated 100,000 species^1^. This algal clade is present in terrestrial, freshwater and marine habitats and each taxon occupies a particular niche^2^. The advantage of these properties of ubiquity and taxonomic diversity were noticed a long time ago. Indeed, the first studies demonstrating the effect of pollution on freshwater diatom communities were over a century ago^3^ and several methods for using diatoms to assess pollution were proposed in the second half of the twentieth century (e.g.^4–6^). Such tools have been used routinely worldwide^7^ in particular to fulfill national or transnational directives (e.g. the Water Framework Directive in Europe^8^ and the National Water-Quality Assessment Program in the USA^9^). Moreover, diatoms often represent an important part of the total biomass in aquatic ecosystems and make a major contribution to global productivity, and therefore cannot be ignored in ecological studies (e.g.^10^).

Classically, diatoms are identified by looking at the gestalt and morphology of their siliceous exoskeleton (frustule) using a microscope. Standard procedure using diatoms as ecological indicators require counting and determining several hundreds of frustules under a microscope^11^. Such procedures are time consuming and, when several thousands of sites need to be monitored, the time and cost of such approaches are substantial (e.g.^12,13^). Moreover, because of the difficulties involved in differentiating between some diatom species, there can be considerable variation between assessment results, even when experienced analysts are involved (e.g.^14,15^).

DNA-barcoding is a taxonomic method that uses a short stretch of DNA to identify species^16^ through comparison with a curated library of reference sequences (i.e. DNA barcodes). Early tests of DNA barcoding to identify animals^16^ and plants^17^ showed a promising ability for non-taxonomists to identify organisms. It was then applied to diatoms^18,19^ and several DNA-markers were evaluated (18S and 28S rDNA, *cox*1, ITS rDNA, *rbc*L). 18S V4 and *rbc*L are the most frequently used markers at present (e.g.^20,21^). When affordable High-Throughput-Sequencing (HTS) methods arrived in 2005, the possibility of using barcoding to analyse environmental samples composed by a mixture of taxa became real. This method, called metabarcoding^22^ uses HTS to sequence environmental samples in order to obtain a large quantity of sequences per run. By comparing each HTS sequence to the barcode reference library, the composition of the environmental community can be ascertained. For diatoms this method was first tested on mock communities made of already-barcoded strains^12^ and then on natural communities from several temperate or tropical rivers^23,24^, lakes^25,26^, marine habitats^27,28^ and even ancient diatom DNA (e.g.^29,30^). When diatoms are the sole focus of the study, a short region with high discrimination capacity is needed and partial *rbc*L (chloroplastic marker) has proven to be suitable (e.g.^12,31^); this is sequenced using specific primers for diatoms. In some other studies the V4 region of 18S (ribosomal marker) has been sequenced; however, its discrimination capacity is slightly lower than that of *rbc*L^12^. On the other hand, the use of generic primers for this region enables a larger range of diversity to be covered than solely diatoms.

The general point raised by all these metabarcoding studies is that an Achilles heel of metabarcoding is the barcode reference library. It must be as comprehensive as possible in order to be able to assign a high proportion of environmental sequences to known taxa, and it requires regular expert curation in order to maintain its quality (i.e. taxonomic assignments, sequences quality and traceability of data and metadata). Several databases curated by experts already exist. Some are general DNA barcoding libraries of protists diversity such as SILVA^32^ and PR2^33^. Others are more focused, e.g. PhytoREF for photosynthetic organisms^34^, PFR2 for planktonic foraminifera (Morard *et al*.^35^), EukRef-Ciliophora for ciliates^36^ and Dinoref for Dinophyceae^37^, although the last database has now been integrated in PR2.

Diat.barcode is a reference library dedicated to diatoms with fine-tuned taxonomy and curation at genus and species level, which is maintained since 2012. This paper describes this open-access barcode reference library and its curation workflow. This library has been used in several ecological studies for rivers and lakes using diatoms (e.g.^12,23,25,26,31,38,39^). Diat.barcode gathers data and metadata for the *rbc*L marker and also, to a lesser extent, for 18S, 28S, cox1 and ITS (only for a few cultures). It is freely accessible through https://www6.inra.fr/carrtel-collection_eng/Barcoding-database. An earlier paper has described the former versions of this database (v1 to v6), it was called R-Syst::diatom^40^. This new paper describes the evolution of the curation procedure, which has been simplified and which is now done collectively by several European experts in diatom taxonomy and phylogeny. Moreover, the name of the database was changed from R-Syst::diatom to Diat.barcode since it moved from a French initiative to an international collaboration. This collective curation ensures a more robust outcome and employs a procedure very similar to that used in EukRef, a community of people with expertise in diverse eukaryotic lineages to curate 18S rDNA data and using phylogenetic methods with the goal of creating a curated reference library of eukaryotes^41^.

Our aim was to produce a curated reference library for diatom metabarcoding. Here we present barcode sources, metadata associated with the barcodes, data curation procedures, and information on data storage and accessibility. Then we show the results of the latest curation of the reference library (version 7) as well as its contents. A description of the release of a ready-to-use database for metabarcoding is also given, namely Rsyst::diatom_rbcl_align_312bp, which is an aligned subset of Diat.barcode.

## Results and Discussion

### Example of the curation of diat.barcode version 7 (February 2018)

The curation of Diat.barcode version 7 was carried out at the start of 2018. Seven different experts worked on it, each focusing on clades corresponding to their specialist knowledge. For instance R. Trobajo and D. Mann curated the clade of the *Nitzschia* s.l. because they have published several papers on this genus (e.g.^42–45^), J. Zimmermann curated the monoraphids because his team published several papers on this group (e.g.^24,46^), M. Pfannkuchen curated newly-described marine diatom sequences, reflecting his laboratory’s interests (e.g.^47–49^) and M. Kahlert the *Fragilaria* genus^50^.

During this process, 703 sequences were curated according to the procedures described above and summarized in Table 1. Among these newly-retrieved sequences, five were removed because the sequence name was too different from the phylogenetic neighbors and no original material was available to check. On the other hand, 698 sequences were kept. Globally, in Diat.barcode version 7, for *rbc*L gene, 605 of the 3482 original names given by the authors of the sequence were modified (i.e. 17%).

**Table 1:** Results of the curation procedure of 23 Feb 2017 corresponding to version 7 of the database.

	Marker	*rbc*L
	Imported sequences in v7 (23 feb 2017)	703
	Sequences in the former version v6 (20 feb 2016)	2784
Curation steps	New sequences having a different id. from the sequence of the same clade	176
	Nomenclatural and taxonomic changes according to peer review papers	33
	Check of photos - modifications of determinations	157
	homogenization of taxonomy/synonymies based on phylogenetic results	1
	Rejected sequences	5
	Sequence in the new version (v7)	3482

Sequences were imported from NCBI (nucleotide database of the National Center for Biotechnology Information) and the TCC (Thonon Culture Collection) between 20 Mar 2016 and 23 Feb 2017. Values in the table give the number of sequences.

### Content of the database

#### Number of sequences and geographical location of the sampling sites

The number of sequences publicly available for *rbc*L is given in Table 2. Sequences from the TCC, the Laboratory of Molecular Systematics of Aquatic plants, Institute of Plant Physiology, Russian Academy of Sciences (curated by Maxim Kulikovsky) and the UK barcoding project (funded by the UK Environment Agency) represent almost 30% of the total. Only 18.7% (210) of the sequences of the TCC and UK barcoding project have so far been deposited in the nucleotide database of the National Center for Biotechnology Information (NCBI), most of these being added to support taxonomic or metabarcoding publications (eg.^51–53^).

**Table 2:** Number of sequences in Diat.barcode version 7 for *rbc*L marker.

	*rbc*L	*rbc*L 312 bp
Total (published in NCBI and in the TCC and UK barcoding project)	3315	167
From TCC and UK barcoding project	955	167

**Figure 1:**
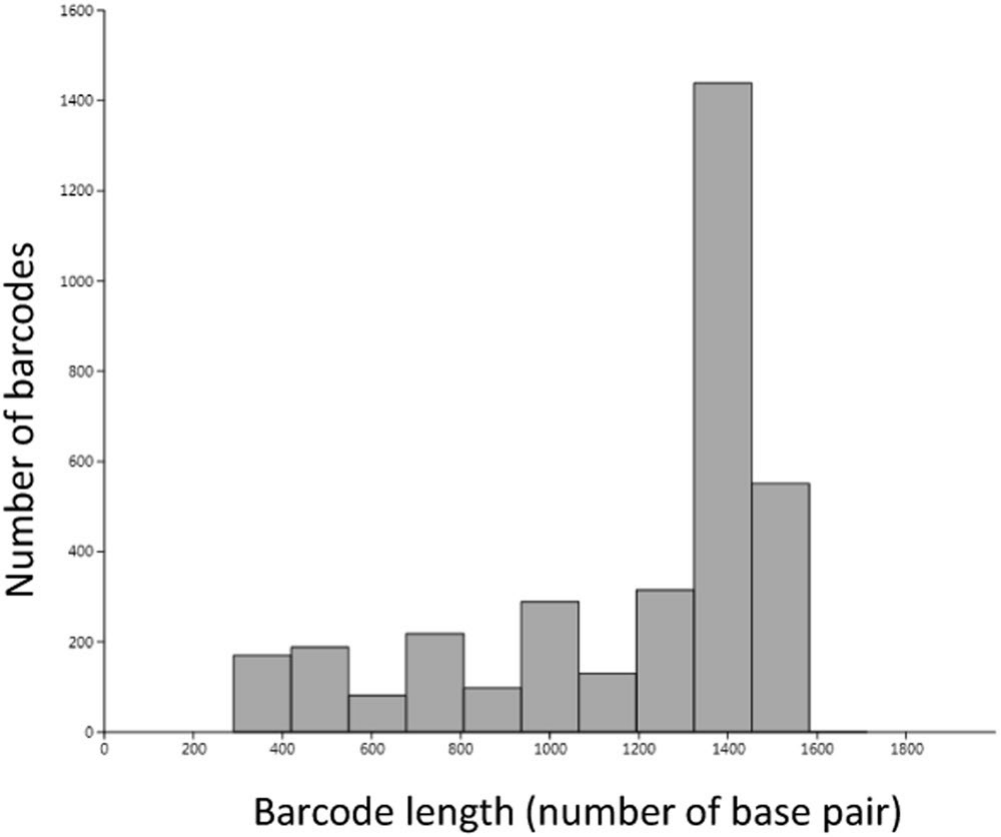
Amount and length of the *rbc*L barcodes present in Diat.barcode version 7 (February 2018).

The lengths of the *rbc*L barcodes in Diat.barcode are given in Fig. 1. Most of the sequences are between 1200 and 1600 bp (sequences longer than 1500 bp include other non-coding portions likely the rbcL-rbcS spacer). A few of them are much shorter (312 bp) and correspond to sequences deposited as part of barcoding studies examining the efficiency of shorter fragments for species identification (e.g. *rbc*L 3 P^54,55^), or as part of metabarcoding studies following^56^.

**Figure 2:**
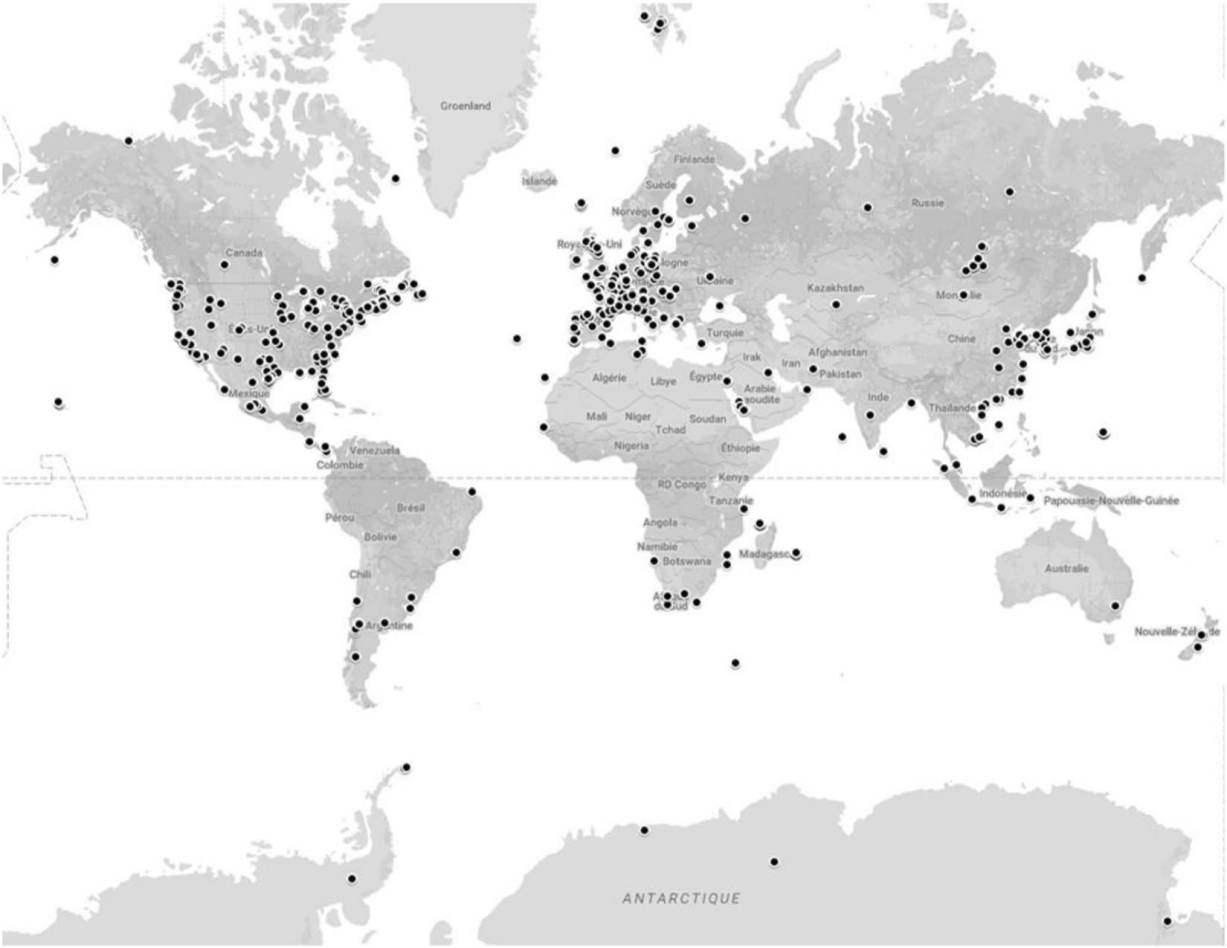
Location of the sampled sites gathered in Diat.barcode (version 7 of February 2018). Map was generated on Google Maps, Map data © 2019 Google (https://www.google.com/maps), accessible at: https://www.google.com/maps/d/u/0/edit?mid = 1Edfju_8jL_lkwSK5pZzBabECXYhMS3y2&ll = -0.7877939911851115%2C0&z = 2

The locations of the sampling sites are given in Fig. 2; they are mostly in Europe and North America, but several are in Asia, including Lake Baikal, Vietnam, Indonesia, Mongolia and Japan (e.g.^57–63^).

#### Number of sequences per diatom class

To show how barcodes are distributed between major structural types in the diatoms, we used the classification given by^64^ which has three classes, Mediophyceae, Coscinodiscophyceae and Bacillariophyceae, and we also retained the Fragilariophyceae as described in^65^; this last class may^64^ or may not be monophyletic but represents a group with similar life-forms and habitat preference (most are attached, non-motile species). We are aware that the taxonomic hierarchy needs considerable modification to make the classes and orders of diatoms monophyletic (e.g.^66^), but we nevertheless use traditional groupings to illustrate the coverage of genera and species of different morphological types. The next version of Diat.barcode will have to integrate the new taxonomy of diatoms (and Eukaryotes) given in^67^. Table 3 gives an overview of the number of barcodes and taxa for each class. The Bacillariophyceae is the most barcoded class, with particularly large numbers of barcodes in the orders Bacillariales and Naviculales. Given that the Coscinodiscophyceae comprises a grade of lineages, some quite species-rich, which diverged from each other early in diatom evolution (e.g.^66^), this group is arguably the least well barcoded.

**Table 3:** Number of *rbc*L barcodes and taxa (species or sub species level) per order in Diat.barcode version 7.

Class	Order	# of barcodes	# of taxa
Bacillariophyceae	Achnanthales	260	55
	Bacillariales	569	114
	Cymbellales	322	90
	Eunotiales	66	12
	Lyrellales	3	3
	Mastogloiales	1	1
	Naviculales	878	263
	Rhopalodiales	31	11
	Surirellales	219	68
	Thalassiophysales	144	39
Coscinodiscophyceae	Asterolamprales	1	1
	Aulacoseirales	25	9
	Chrysanthemodiscales	1	1
	Corethrales	2	1
	Coscinodiscales	24	14
	Leptocylindrales	3	2
	Melosirales	28	10
	Paraliales	76	6
	Rhizosoleniales	16	9
Fragilariophyceae	Ardissoneales	4	4
	Climacospheniales	1	1
	Cyclophorales	5	4
	Fragilariales	333	81
	Licmophorales	10	8
	Protoraphidales	1	1
	Rhabdonematales	3	2
	Rhaphoneidales	8	5
	Striatellales	13	10
	Tabellariales	11	3
	Thalassionematales	8	4
Mediophyceae	Anaulales	1	1
	Biddulphiales	14	11
	Chaetocerotales	29	15
	Cymatosirales	42	16
	Hemiaulales	10	9
	Lithodesmiales	19	6
	Thalassiosirales	212	86
	Toxariales	2	2
	Triceratiales	83	32

For the Bacillariophyceae, many sequences were recently published for *Achnanthidium* and *Planothidium* by^68^ and^46^ (this later publication was part of the German Barcode of Life 2 -GBOL2- Diatoms project funded by the German Ministry for Education and Research -BMBF-). Many also come from the UK-barcoding project. Similarly, a number of newly sequenced strains from the genus *Nitzschia* have been added thanks to studies in marine coastal environments (^69,70^ and^45^) and from the UK-barcoding project. A major taxonomic study of the Surirellales and Rhopalodiales orders also resulted in many new taxa being sequenced, leading to a major reevaluation of the taxonomy, with creation of new genera and transfers of taxa^71^. Finally, barcodes for 21 taxa that are important for ecological assessment but which have not yet been sequenced (either from cultured strains or from single cells) were obtained directly using HTS data from environmental samples (^56^), as part of a project funded by the French Biodiversity Agency (Agence Française pour la Biodiversité) in 2017 and 2018.

## Conclusions

A barcode reference library is like a ‘molecular dictionary’, where each molecular sequence is matched up with its organism. Therefore, a comprehensive and well curated reference database – with high quality sequences and up-to-date, correct taxonomy – is a key factor in both phylogenetic as well as metabarcoding studies (e.g.^20,33,36,37,41^). The traceability and availability of metadata (sampling site, isolation protocols, pherograms, vouchers, slides, DNA, photos…) and the accessibility of physical vouchers for the barcodes (culture, raw material, slides, DNA…) are necessary for scientific studies. This point is so important that European diatom experts (FR, DE, HR, HU, UK, ES, CZ, BE…) have worked with the European Standardization Committee to prepare a technical report^72^ as a first step towards standardization of this process. Algal barcoding libraries such as Diat.barcode and Algaterra already fulfill the requirements of this report.

## Methods

### Data sources

Data sources used to fill Diat.barcode come from:

- barcoded strains of the Thonon Culture Collection (TCC)^73^; many of these sequences have not yet been published formally in peer-reviewed journals and are not deposited in GenBank;
- barcoded strains of the UK-Barcoding project funded by the UK Environment Agency^31^; similarly, many of these sequences have not yet been published;
- barcoded strains of the Laboratory of Molecular Systematics of Aquatic plants Institute of Plant Physiology Russian Academy of Sciences (curated by Maxim Kulikovsky); many of these sequences have not yet been published formally in peer-reviewed journals;
- diatom sequences published in the nucleotide database of the NCBI.

#### Barcoded strains and samples of the TCC

The UMR-CARRTEL is a research unit of the French National Institute for Agricultural Research (INRA). It has maintained the TCC since 1968^73^. 881 monoclonal strains of freshwater microalgae are registered (486 are alive at the moment of the manuscript submission), among which 543 are diatoms. For each culture, DNA extracts and raw material are kept in the UMR-CARRTEL. Moreover, for diatoms, at least one permanent slide of cleaned frustules (mounted using Naphrax) is kept for each strain, along with nitric acid-treated material (in a vial). This material is accessible for subsequent studies. The strains are available on request through a website dedicated to the collection (http://www6.inra.fr/carrtel-collection_eng/). Each strain is sequenced at least for *rbc*L.

In addition to cultures, 66 uncultured samples, which were sequenced using HTS, have been preserved and are kept in the TCC. Indeed, it is possible to relate environmental sequences to the target species observed (and determined) by microscopy and, consequently identify them with high reliability^56^. In such cases, only a short fragment of *rbc*L is sequenced (312 bp with Illumina Miseq). This method allows to obtain sequences from diatom species that are difficult to culture. For these uncultured samples, permanent slides and raw material are kept (in ethanol) in the TCC.

All information about these strains and uncultured samples, such as sampling location, isolator, barcode (including type of barcode, amplified region, primer used, protocols, sequencing technology), phenotypic data, photos, associated research programs (for sampling and sequencing) and its taxonomic affiliation are available at https://www6.inra.fr/carrtel-collection_eng/Barcoding-database.

#### Barcoded strains of the UK-barcoding project funded by the UK environment agency

This project^31^, funded by the UK Environment Agency, England’s environmental regulator, aimed to develop a DNA metabarcoding approach to ecological assessment based on diatoms using HTS of a fragment of the *rbc*L gene^31^. It aimed to ensure continuity with microscopical methods while, at the same time, complying with the EU Water Framework Directive, which refers to ‘taxonomic composition’. The twin foundations for this study were a calibration dataset of samples, analyzed by both microscopy and HTS approaches, along with a reference database of *rbc*L DNA barcodes which link to Linnaean taxonomy. The samples spanned a wide range of ecological status encountered throughout the UK.

Individual cells of diatoms were isolated by micropipette or by streaking on 2–3% agar plates. A genus-level identification of living cells was made using an inverted microscope (x40). This enabled interesting cells to be selected and transferred to a synthetic medium to grow these cells for subsequent morphological (photos) and molecular (DNA extraction, PCR, Sanger sequencing) analyses. A total of 987 unialgal cultures were obtained from samples collected from 60 locations in England and Scotland. DNA was extracted and sequenced from these cultures. Information about these strains (sampling site location, isolators, barcode, photos) and their taxonomic affiliation are available at https://www6.inra.fr/carrtel-collection_eng/Barcoding-database. DNA has been retained in the Edinburgh DNA (EDNA) bank at the Royal Botanic Garden Edinburgh (E). Voucher slides (mounted in Naphrax) and material are kept in the diatom herbarium at E.

#### Barcode strains of the laboratory of molecular systematics of aquatic plants of institute of plant physiology russian academy of sciences

A total of 3218 monoclonal strains of freshwater, brackish and marine diatoms from almost all parts of Russia as well as Mongolia, Vietnam, Indonesia, Arctic zone, Spain, Japan, Ethiopia are registered (1678 are alive at the moment of the manuscript submission). For each culture, DNA extracts and raw material are kept in the Institute of Plant Physiology. At least one permanent slide of cleaned frustules (mounted using Naphrax) is kept, along with nitric acid-treated material (in a vial). This material is accessible for subsequent studies. These strains are available on request. Each strain is sequenced at least for 18S and *rbc*L. All information about these strains, such as sampling location (georeferenced on a google map), isolator, barcode (including type of barcode, amplified region, primer used, protocols, sequencing technology), phenotypic data, photos, associated research programs (for sampling and sequencing) and its taxonomic affiliation are available or will be available for the next version of Diat.barcode (version 9) in 2020 at https://www6.inra.fr/carrtel-collection_eng/Barcoding-database.

#### Nucleotide database of NCBI

The National Center for Biotechnology Information (NCBI), in the USA, maintains a webserver that collects and provides molecular data and software; these data are publicly accessible via the GenBank database (^74^, http://www.ncbi.nlm.nih.gov/genbank). Initially, all 18S (including V4-region of 18S) and *rbc*L nucleotide sequences of diatoms (freshwater and marine) available in GenBank’s main collection (CoreNucleotide), whatever their length and their quality, were assessed. We limited the search to these markers because they proved to be effective for species identification (e.g.^51,54,75,76^) and showed the best results for metabarcoding^12,23,24^. Sequences for 28S rDNA, ITS and *cox*1 were not gathered in the database and, since March 2017 (version 5), 18S sequences are not integrated anymore for practical reasons. Indeed, given the increasing number of diatom sequences available on GenBank and the increasing size of Diat.barcode database, we have decided to work solely on *rbc*L sequences as curating both makers (18S and *rbc*L) was too much work and other initiatives are underway to curate 18S^41^.

Sequences are retrieved regularly (every 6 months) using the following keywords on the Nucleotide Advanced Search Builder: “(*rbc*L) and (diatom OR Bacillariophyta)”. Additionally, a publication interval in NCBI is indicated in the Advanced Search Builder: the earliest date corresponds to the previous Diat.barcode update and the most recent to the current date. Diat.barcode is thus updated every 6 months or every year. Unlike 18S, the number of environmental (cloned) sequences available for *rbcL* is low compared to the number of sequences derived from cultured strains. Hence we do not search NCBI using BLAST.

### Data curation

Sequences that can potentially be integrated into Diat.barcode are from different sources (e.g. GenBank, national barcoding projects, etc…). There are two important drawbacks to consider when gathering new sequences into Diat.barcode and dealing with these is crucial for curation.

First, the earliest data were produced in 1998 and there have been substantial changes in our understanding of diatom taxonomy over the period since then. Moreover, the identifications and taxonomic skills vary between the different authors of the data and have also evolved over time. This means that taxonomy needs to be harmonized before these data are gathered into Diat.barcode.

Second, the quality of the sequences can be suboptimal for correct taxonomic affiliation. Such sequences of low quality are not integrated in Diat.barcode.

These two drawbacks underline the importance of curating the data in order to keep a high quality reference library. The first step of the curation is therefore to check the quality of the sequences. The second step is to curate the species assignment of the strains (from now on “names”): the aim is to achieve, for genetically similar sequences, congruence in their names. The name must be correct according to the most recent taxonomical developments which are themselves subject to expert evaluation. However, as diatom taxonomy is developing rapidly, there will be cases where only a consensus for practical use can be made and solutions regarding the correct name will have to await further studies. In any case, if the original name given by the authors of the sequence was changed during the curation procedure, the traceability of the original name is kept in the database.

This data curation is carried out in two main steps which are very similar to the method used in EukRef^41^:

- The 1st step checks the quality and length of sequences and inserts them in a multiple alignment
- The 2nd step, uses a constrained phylogenetic analysis to check that similar sequences (for instance belonging to the same clade) are assigned to the same name. If not, the names are checked through a taxonomic curation procedure.

**Figure 3:**
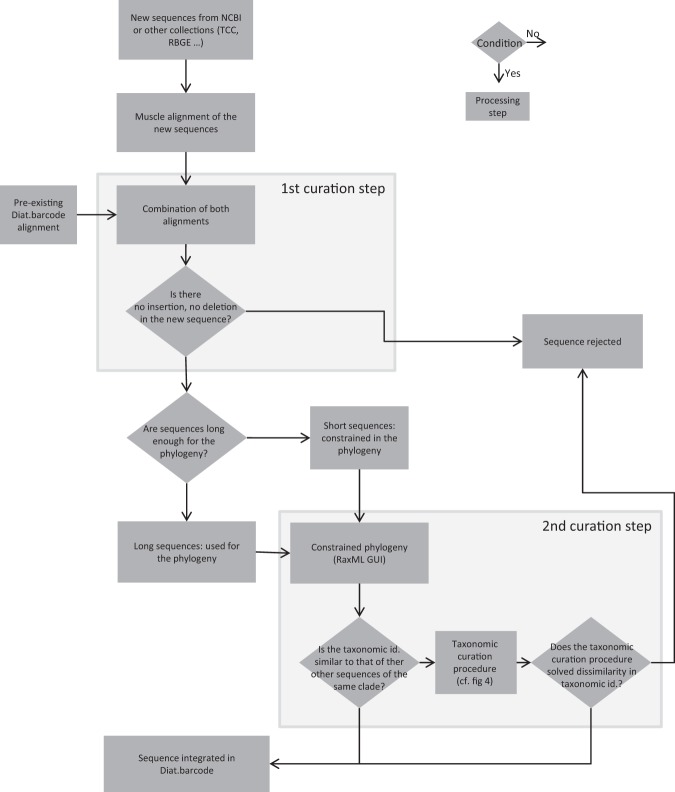
General flowchart of the curation and integration of new sequences in Diat.barcode library. Taxonomic curation procedure is detailed in Fig. 2. Diamonds are conditions, the arrow from the bottom point of the diamond corresponds to « Yes », the arrow from the right point of the diamond corresponds to « No ». Rectangles are processing steps.

**Figure 4:**
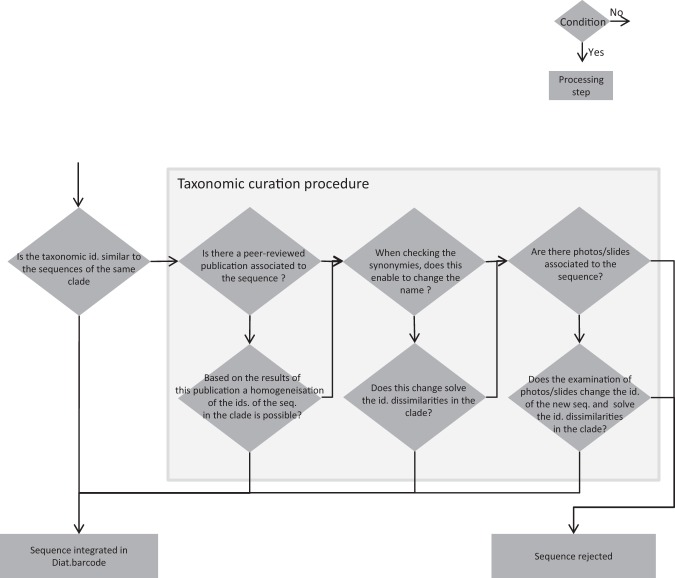
Flowchart of the taxonomic curation procedure. Diamonds are conditions, the arrow from the bottom point of the diamond corresponds to « Yes », the arrow from the right point of the diamond corresponds to « No ». Rectangles are processing steps.

Figure 3 gives an overview of the general workflow of the curation procedures. Figure 4 gives details on the taxonomic curation procedure listed in Fig. 3.

#### First curation step: checking for sequence quality

A multiple alignment is carried out with the new sequences, usually using Muscle in Seaview^77^. In parallel, a multiple alignment for *rbc*L of the entire Diat.barcode library has been maintained for several years. This alignment has been progressively updated year after year and show no insertions nor deletions since *rbc*L is a coding region, unless insertions or deletion are multiples of 3 nucleotides (until now no frameshifts were encountered so far). The alignment with the new sequences is then added to the alignment of the entire Diat.barcode library. If insertions or deletions appear when adding the new sequences, then the sequences with these insertions or deletions are removed (see box “1^st^ curation step” in Fig. 3). Sequences with ambiguity codes are removed.

#### Second curation step: taxonomic curation based on a phylogenetic analysis

Construction of a constrained phylogeny: the objective here is to construct a constrained phylogeny that can be used to assess the taxonomic assignment of the sequences (new and already in Diat.barcode library). We made a constrained phylogeny in order to place the shortest sequences in a phylogeny built with the longest sequences. In the alignment obtained in the first curation step (and which includes new sequences and sequences from Diat.barcode library), sequences are ranked (see diamond “Sequences long enough for the phylogeny” in Fig. 3) into:

- long sequences, starting before nucleotide position 250 of the alignment and ending after nucleotide position 1129; these sequences are used to construct a phylogeny including 879 alignment positions;
- short sequences, starting after nucleotide position 250 and/or ending before nucleotide position 1129; these sequences are not used for the phylogeny construction and will be placed afterwards, using a phylogeny constrained by the topology of the tree inferred only with long sequences.

The short sequences are marked with an “*” at the beginning of their sequence name (it is important to identify such sequences to ensure that they are not used to construct the phylogeny). The constrained phylogeny is made using raxmlGUI^78^. In the raxmlGUI menu, the option “Enforce constraint” is selected, followed by “Define topological constraint”, and finally the short sequences are used as “constrained taxa”. Then a ML + rapid bootstrap with 100 + GTR Gamma model is launched (such calculation takes 3 to 4 days’ calculation using 2 threads on a computer equipped with 8 Intel®Xeon® CPU E5-1620 3.60 ghz).

Taxonomic curation: The taxonomic curation procedure (Fig. 4) focuses only on the clades bearing new sequences (long or short sequences). The new sequences are marked with an “@” at the beginning of their sequence name in order to recognize them easily in the phylogeny. New short sequences are therefore prefixed “*@”.

Here the overall objective is to check that for each well-supported monophyletic clade (bootstrap values above 50%) phylogenetic neighbor sequences of the new sequences « @ » have homogeneous names. If they are not homogeneous, several verifications are done as explained below:

i. Check if a peer-reviewed publication is associated with the new sequence. If this is the case, the results of the publication can be used to assess if the names of the new and/or old sequences can be homogenized for the clade containing them. Depending on the case, homogenization might be achieved at the species level or, if this is not possible, at the generic level or even higher level. This homogenization can take place in any of several points (i, ii of iii). If these modifications result in a clade with homogenized names, the new sequence(s) is (are) kept.
ii. If no such peer-reviewed publications are available, the synonymies of the names of the sequences inside the clade are checked. We use of the online taxonomic catalogue DiatomBase at diatombase.org^79^ which integrates the former Catalogue of Diatom Names^80^. The online taxonomic catalogue AlgaeBase can be used as well^81^ only if DiatomBase lacks the information looked for. If this results in homogenized taxonomy, the sequence and the new taxonomic names are kept.
iii. If this is not the case, we check if photos/slides are associated with the sequence(s). There are several websites from which photos of strains are available, for instance:

- AlgaTerra accessible at http://www.algaterra.org is maintained by the Botanic Garden and Botanical Museum Berlin-Dahlem (Germany, W.-H. Kusber, R. Jahn, N. Abarca, O. Skibbe & J. Zimmermann);
- Protist central accessible at http://www.protistcentral.org/ is maintained by the Texas Natural Science Center of the University of Texas (USA, E. Theriot);
- the website of Papanin Institute for Biology of Inland Waters Russian Academy of Sciences http://ibiw.ru/index.php?p = project/algo/WDCM602/mgmt_class&id = 7&lang = en (Russia, M Kulikovsky);
- Bold accessible at http://www.boldsystems.org/ ^82^ is maintained by the University of Guelph.

If the re-examination of the photos/slides results in a change in the name associated with the sequence that better fits its position in the *rbc*L-based phylogenetic tree, the sequence and its new taxonomic name is kept. If the taxonomic name is still different after checking photos/slide, the sequence is rejected.

Each time a modification of the name is made, a record of all changes is kept. The original name given by the author of the sequence is kept in Diat.barcode files.

### Data access

The websites giving access to download the data are accessible at https://www6.inra.fr/carrtel-collection_eng/Barcoding-database.

For each strain, the following information is given, if available: sampling site information (name and coordinates), type of habitat, strain code given by the laboratory, name of the project which funded the field sampling, the laboratory responsible for field sampling, DNA extraction, PCR, sequencing, and the dates of the different steps. A species name is given to each strain, except in a few cases where only the genus is given. In addition, the taxonomic affiliation is given up to the kingdom, following the hierarchical system given in Algaebase^81^. For molecular criteria, the database gives the type of marker (18S, 28S, ITS2, cox1, *rbc*L), the primers used for sequencing and PCR. Protocols for DNA extraction and PCR are also given and the laboratory responsible for the sequence is named. Phenotypic information is given (including average species dimensions, chloroplast shapes and number, life-form, ecological class, sensitivity values for widely-used biotic indices). Photos (living material or/and cleaned frustules or valves) of the strains coming from the TCC^73^, the UK-Barcoding project funded by the UK Environment Agency^31^ and from Maxim Kulikovsky, are also downloadable and the web links are given at https://www6.inra.fr/carrtel-collection_eng/Barcoding-database.

#### A ready-to-use database for metabarcoding: Rsyst::diatom_rbcl_align_312bp

As stated earlier, the use of HTS methods and metabarcoding is getting more and more common to study the composition of environmental samples. Current HTS platforms such as Illumina MiSeq enable fragments of around 300 bp to be sequenced. The most common barcode used today for diatoms is a 312 bp long stretch of the *rbc*L marker (e.g.^13,25,27,31,38,56^). Therefore, the ability to extract this particular region from Diat.barcode is necessary. The authors of this paper, in particular V. Vasselon, have made this extraction from the multiple alignment of the different sequences present in Diat.barcode. Some species, which can be distinguished on the basis of full length of *rbc*L (~1500 bp) sequences, can have identical sequences when this 312 bp barcode is used. This means that a further curation procedure is required. This procedure, called “Rsyst::diatom_rbcl_align_312 bp”, is described below:

First, the 312 bp *rbc*L barcode is extracted from the full Diat.barcode database *rbc*L alignment (by reference to the positions of the Diat_rbcL_708F^83^ and R3^84^ primers). Sequences with ambiguities (N), homopolymers longer than 8 and sequences shorter than 312 bp are removed from the database. The resulting sequences are dereplicated into Individual Sequence Units (ISUs) in order to identify taxa sharing identical DNA sequences in the 312 bp *rbc*L barcode region. If necessary, we harmonize the taxonomy between all taxa found in each single ISU. Finally, only ISUs are retained in the database, each represented by one taxon ID and one DNA sequence. The resulting ISUs database is assigned to itself using the Mothur assignment algorithm (classify.seqs command). The new taxonomy is compared with the expected assignments to evaluate potential sources of bias (erroneous taxonomic names, taxa impossible to differentiate,…). If necessary, ambiguous sequences are removed from the database or the taxonomy is adjusted. Finally, we harmonize potentially conflicting names (e.g. “aff.” and “cf.” are removed; “Nanofrustulum_sp._SZCZCH285” transformed into “Nanofrustulum_sp.”).

The resulting files are a “.fasta” file which contains the *rbc*L 312bp DNA sequences and a “.txt” file which contains the corresponding taxonomy and the sequence identifier common to both files. The sequence identifier is composed of an accession number (also present in the Diat.barcode library) and the original name given by the author of the sequence (eg: TCC679-Rbcl-1|Achnanthidium_pyrenaicum). The text file gathers the curated taxonomical information from empire to species level.

For instance, for TCC679-Rbcl-1|Achnanthidium_pyrenaicum the taxonomy is Eukaryota; Chromista; Chromobiota; Bacillariophyta; Bacillariophyceae; Achnanthales; Achnanthidiaceae; Achnanthidium; Achnanthidium_minutissimum. In this case, Achnanthidium_pyrenaicum is the original species name given by the author and Achnanthidium_minutissimum is the curated species name that will be used in metabarcoding. This database has been curated for a specific use with filtering procedures to meet our own needs (especially for use in metabarcoding for diatom based ecological assessment in rivers and lakes) and is provided on this basis. The original database, Diat.barcode, is the reference and can be curated differently to meet different requirements.
